# Immune Dysregulation in Acute SARS-CoV-2 Infection

**DOI:** 10.20411/pai.v7i2.537

**Published:** 2023-02-20

**Authors:** Lauren Grimm, Chinyere Onyeukwu, Grace Kenny, Danielle M. Parent, Jia Fu, Shaurya Dhingra, Emily Yang, James Moy, PJ Utz, Russell Tracy, Alan Landay

**Affiliations:** 1 Department of Internal Medicine, RUSH University Medical Center, Chicago, IL; 2 Centre for Experimental Pathogen Host Research, University College Dublin, Ireland; Department of Infectious Diseases, St Vincent’s University Hospital, Dublin, Ireland; 3 Department of Pathology and Laboratory Medicine and Department of Biochemistry, University of Vermont Larner College of Medicine, Burlington, VT; 4 Division of Immunology, Department of Medicine, Stanford University, Stanford, CA

**Keywords:** SARS-CoV-2, adaptive immunity, spike antibody, cytokines, autoantibodies

## Abstract

**Introduction:**

Neutralizing antibodies have been shown to develop rapidly following SARS-CoV-2 infection, specifically against spike (S) protein, where cytokine release and production is understood to drive the humoral immune response during acute infection. Thus, we evaluated the quantity and function of antibodies across disease severities and analyzed the associated inflammatory and coagulation pathways to identify acute markers that correlate with antibody response following infection.

**Methods:**

Blood samples were collected from patients at time of diagnostic SARS-CoV-2 PCR testing between March 2020-November 2020. Plasma samples were analyzed using the MesoScale Discovery (MSD) Platform using the COVID-19 Serology Kit and U-Plex 8 analyte multiplex plate to measure anti-alpha and beta coronavirus antibody concentration and ACE2 blocking function, as well as plasma cytokines.

**Results:**

A total of 230 (181 unique patients) samples were analyzed across the 5 COVID-19 disease severities. We found that antibody quantity directly correlated with functional ability to block virus binding to membrane-bound ACE2, where a lower SARS-CoV-2 anti-spike/anti-RBD response corresponded with a lower antibody blocking potential compared to higher antibody response (anti-S1 r = 0.884, *P* < 0.001; anti-RBD r = 0.75, *P* < 0.001). Across all the soluble proinflammatory markers we examined, ICAM, IL-1β, IL-4, IL-6, TNFα, and Syndecan showed a statistically significant positive correlation between cytokine or epithelial marker and antibody quantity regardless of COVID-19 disease severity. Analysis of autoantibodies against type 1 interferon was not shown to be statistically significant between disease severity groups.

**Conclusion:**

Previous studies have shown that proinflammatory markers, including IL-6, IL-8, IL-1β, and TNFα, are significant predictors of COVID-19 disease severity, regardless of demographics or comorbidities. Our study demonstrated that not only are these proinflammatory markers, as well as IL-4, ICAM, and Syndecan, correlative of disease severity, they are also correlative of antibody quantity and quality following SARS-CoV-2 exposure.

## INTRODUCTION

The virus known as severe acute respiratory syndrome coronavirus 2 (SARS-CoV-2) initially emerged in Wuhan, China, in December 2019 and eventually led to the largest global pandemic in over a century. Coronavirus disease 2019 (COVID-19) has caused significant morbidity and mortality, especially among individuals over the age of 65 and those with preexisting medical conditions, including hypertension, diabetes, and cardiovascular disease, among others. As of November 1, 2022, the United States has experienced over 1,000,000 deaths due to COVID-19, with over 98.1 million people known to have been infected [1]. While extraordinarily rapid scientific advances toward understanding SARS-CoV-2 have been made since its initial discovery, there is still a long way to go in understanding the complete pathophysiologic and immunologic picture of COVID-19 disease.

To date, our best understanding of the adaptive immune response following COVID-19 comes from serologic analysis of previously confirmed cases. Studies have shown that the vast majority of SARS-CoV-2 infected individuals seroconvert within 5 to 15 days post-symptom onset, with 90% seroconversion by day 10 [2, 3]. Similarly, antibodies have been shown to develop rapidly following SARS-CoV-2 infection, specifically against spike (S) protein on the cell surface, nucleocapsid (N) protein, and membrane glycoprotein (M), where antibodies exhibit little to no somatic hypermutation [4]. An increased antibody response has been shown to be associated with an increase in and/or persistence of viral load [5]. Similarly, increasing COVID-19 disease severity has been shown to be associated with increased antibody response, where asymptomatic and mild disease has been associated with a lower but effective peak in IgG and IgA titer concentration compared to that seen in individuals with moderate and severe disease, whose responses are much more robust [6–8].

Cytokine release and production of a proinflammatory microenvironment is understood to drive the immune response during acute infection. Cytokine release syndrome, or cytokine storm, is a well-described driver of inflammation and severe disease in patients with COVID-19, especially among those with preexisting, comorbid conditions [9]. Previous studies have shown that increased interleukin (IL)-6 and tumor necrosis factor alpha (TNFα), along with T helper 1-specific cytokines such as interferon-induced protein 10 (IP-10), are associated with severe COVID-19 response and mortality [10–12]. Similarly, specific cytokines such as IL-6, IL-8, and IL-10 have all been found at higher levels in blood derived from SARS-CoV-2-infected patients who experienced acute respiratory distress syndrome (ARDS) [13]. While more data linking cytokine levels to COVID-19 disease severity have recently emerged, studies related to the acute phase proinflammatory microenvironment and SARS-CoV-2 antibody response are limited. Therefore, to better understand the quantitative and qualitative humoral immune response to SARS-CoV-2, we evaluated the quantity and function of antibodies across disease severities and analyzed the associated inflammatory and coagulation pathways to identify acute markers that correlate with antibody response following infection.

## METHODS

### Study Samples

Blood samples were collected from patients at time of diagnostic SARS-CoV-2 PCR testing between March 2020 and November 2020 at Rush University Hospital. The samples were stored in a biorepository for future analysis. Of the longitudinal samples, plasma samples were subsequently collected between 1 week and 4 months after initial COVID-19 presentation. In addition, pre-pandemic samples collected between 2004 and 2014 were used as negative controls.

Plasma samples were grouped by disease severity based on the CDC COVID-19 Clinical Spectrum of Disease classification: (1) Pre-Pandemic Negative Controls; (2) PCR-Negative Mild Disease: samples from patients presenting to Rush University Hospital with COVID-19 symptoms but who later tested negative for SARS-CoV-2 by PCR; (3) PCR-Positive Mild COVID-19 Disease: samples from patients presenting to Rush University Hospital with COVID-19 symptoms and later tested positive for SARS-CoV-2 by PCR, not requiring hospitalization; (4) PCR-Positive Moderate COVID-19 Disease: samples from patients presenting to Rush University Hospital with COVID-19 symptoms who required inpatient services due to evidence of lower respiratory tract infection, but did not require oxygen supplementation, and who tested positive for SARS-CoV-2 by PCR; (5) PCR-Positive Severe COVID-19 Disease: samples from patients presenting to Rush University Hospital with COVID-19 symptoms requiring inpatient services, including oxygen supplementation and/or ICU admission, and who tested positive for SARS-CoV-2 by PCR.

### Measurement of Antibody Response

Plasma samples were analyzed using the MesoScale Discovery (MSD) Platform 9-plex Assay. The MSD COVID-19 Serology kit measures antibodies that target antigens found on the surface of SARS-CoV-2; spike, Receptor Binding Domain (RBD) and nucleocapsid, as well as spike antigens found on the surface of other coronaviruses implicated in human pathologies of varying severities, including SARS-CoV-1, 229E, OC43, NL63, and HKU1.

MSD provides plates with target antigens coated onto the surface of individual carbon spots. Next, diluted human plasma or serum is introduced onto the surface of the spots and incubated. Antibodies found in human plasma or serum bind to their target antigens. The plate is then washed to remove unbound protein. MSD SULFO-TAG labeled anti-human IgG antibodies are then added to the sample wells and incubated. Finally, the plate is washed again to remove unbound proteins, and a proprietary reading buffer is added. The plate is then loaded onto MSD Quickplex 120, where an electrical current is applied to each well, creating a chemiluminescent reaction that causes the SULFO-Tag to emit light. A high-resolution charge-coupled device (CCD) camera and lens system detects the light emitted, and the signal is back fitted to a calibrator that is standardized against WHO International Standard NIBSC 20/136 (Table 1).

**Table 1.** MSD Platform Analyte Detectable Range and Quality Control

Panel	Catalog Number	Analyte	Calculated Min Detectable Concentration (AU/mL)		Calibrator Range (AU/mL)	Dilution Factor	Detectable Assay Range (AU/mL)	QC CV% Range	QC CV% Average	Comment
Coronavirus Serology	K15369U	Spike	0.0035	3.52	0.004272 – 700.00	1,000	4.272 – 700,000	1.63% – 12.46%	6.08%	Quantitative IgG Serology: Calibrator in Arbitrary Units/mL (AU/mL)
		RBD	0.0024	2.36	0.001831 – 300.00	1,000	2.36 – 300,000	5.14% – 55.36% ^*^	22.09%	
		Nucleocapsid	0.0058	5.75	0.004883 – 800.00	1,000	5.75 – 800,000	0.06% – 6.83%	3.05%	
		229E	0.0027	2.73	0.0018 – 300.00	1,000	1.83 – 300,000	0.03% – 7.16%	2.79%	
		CoV-1 Spike	0.0049	4.91	0.0003 – 50.00	1,000	4.91 – 50,000	2.15% – 26.21% ^*^	8.77%	
		CoV-2 NTD	0.00018	0.18	0.000061 – 10.00	1,000	0.18 – 10,000	1.21% – 62.51% ^*^	16.00%	
		HKU1	0.0965	96.47	0.0018 – 300.00	1,000	96.47 – 300,000	2.39% – 14.65%	6.39%	
		NL63	0.0031	3.08	0.0003 – 50.00	1,000	3.08 – 50,000	2.56% – 8.86%	4.91%	
		OC43	0.1010	101.04	0.0031 – 500.00	1,000	101.04 – 500,000	1.52% – 7.16%	4.49%	

Validation data was performed by the manufacturers of the MesoScale Discovery (MSD) Platform. Validation data performed by the authors included comparison of the data collected from the MSD against other methods, including the Luminex SARS-CoV-2 panel, Quanterix Spike Assay, UVM Homebrew Assay(s), and some ELISAs, to ensure accuracy and reproducibility. Other validation data performed by the authors to ensure reproducibility and validity of study data include PCR-positive/negative agreement, precision and accuracy, and lot-to-lot validation.

* High CV% Driven by pre-pandemic QC replicate(s); replicates low on calibrator curve

### ACE2 Blocking Assay

SARS-CoV-2 neutralizing IgG antibodies were assessed via the MSD V-Plex SARS-CoV-2 Panel 2 (ACE2) Kit in accordance with the kit instructions (Catalog K15386U). The V-Plex COVID-19 ACE2 Neutralizing Kit(s) measure antibodies that block the binding of angiotensin-converting enzyme 2 to the SARS-CoV-2 spike and RBD antigens. MSD provides MSD multi-spot 96-well plates that are coated with SARS-CoV-2 spike and RBD antigens, on unique independent carbon fiber spots within each well. Calibrator, controls, and human plasma, serum or other body fluids are introduced onto the plate. Human plasma is diluted 100x prior to the addition of plasma to the plate. After a 1-hour incubation, ACE2 detection solution is added to each well.

The samples are NOT washed after the 1-hour incubation. The ACE2 detection solution is ACE2 protein that has been labeled with MSD SULFO-Tag detection solution. The ACE2 detection solution binds to any available antigen on each coated spot in each well. After another 1-hour incubation, the plate is washed to remove any unbound proteins. MSD Gold Read Buffer B is then added to each well and read on an MSD Analyzer QuickPlex SQ 120. On board the analyzer, an electrical signal is applied to each well of the plate, which causes a chemical reaction that emits light proportional to the quantity of measured analyte on each unique spot and in each well. For the neutralizing assay 2 data points are available for analysis. First, the quantity of SARS-CoV-2 neutralizing IgG can be quantified by back calculating against the standard curve using 4-parameter logistic regression to obtain a result in neutralizing activity in ug/mL. The second method is percent inhibition, which is employed for this paper. Percent inhibition is calculated utilizing the following formula: 1 – (Average Sample ECL Signal/Average ECL Signal of the blank)*100.

### Measurement of Cytokine Response

IFNγ, IL-1β, IL-4, IL-6, IL-10, IP-10, TNF-α, and Syndecan 1 were all run on an MSD U-Plex 8 analytes multiplex plate. U-Plex assays have biotinylated capture antibodies specific for each analyte of interest. The biotinylated antibodies are coupled to proprietary U-Plex linkers that are unique to each spot on a 10-spot plate. Calibrator, control, and human plasma are then introduced to each well and incubated at room temperature. After the incubation period, unbound proteins, molecules, and plasma are washed and removed from the plate wells. Detection antibodies that are conjugated to the electro-chemiluminescent label SULFO-tag are then added, which completes the immunoassay sandwich. After an incubation period, unbound detection antibody is washed and removed from the plate wells. Read buffer is added, and the U-Plex plate is loaded into the MSD instrument where a voltage applied to the plate electrodes causes the capture labels to emit light. The instrument measures the intensity of the emitted light, which is proportional to the amount of analyte(s) present in the sample. The concentration of each analyte is back calculated based on a calibration curve to determine the quantitative measure of each analyte in the sample.

### Measurement of Soluble ACE2, Cathepsin-L, ICAM, and D-Dimer

Human ACE2 levels were quantified using the Simoa Quanterix HD-X analyzer. R&D Systems Human ACE2 Antibody 0.25 mg/mL (catalog number MAB9331) was covalently coupled to 500 uL batch size Quanterix 488 nM beads (catalog number 103526) using the Simoa Homebrew Assay Starter Kit (Catalog 101351). After antibody-bead conjugation was completed, the Simoa Quanterix HD-X analyzer was programmed to run a fully automated 3-step digital assay in the following manner:

First, antibody-coated paramagnetic beads are incubated with calibrators of known concentration and specimens of unknown concentrations. The calibrator used for this assay is R&D Systems Recombinant ACE2 Protein, carrier free (Catalog Number 933-ZN-010).

After undergoing a wash cycle, the beads and captured analyte are incubated with biotinylated detection antibody. The detection antibody used for this assay is R&D Systems Human ACE2 Biotinylated Antibody, (catalog BAF933).

After undergoing a second wash cycle, the beads/analyte/biotinylated antibody complex is incubated with the enzyme conjugate, streptavidin β-galactosidase (SBG) & substrate, β-D-galactopy-ranoside (RGP). The bead complex with captured analyte is introduced to a proprietary disc with micro-wells large enough for 1 bead complex, via a slight vacuum. Oil is delivered to the array via the same channel, removing excess beads from the surface of the array and sealing the loaded wells. The instrument camera images the sealed wells, capturing the fluorescence emitted by the enzymatic product of the labeled immunocomplexes. The instrument analyzes the image, determines the average enzymes/bead (AEB), and generates a calibration curve and sample concentration back calculated via 4-parameter logistic regression using the values from the calibration curve.

Human Cathpesin L levels were quantified using the Simoa Quanterix HD-X analyzer. R&D Systems Human Cathepsin L Antibody 0.20 mg/mL (part number 842811, catalog number DuoSet DY952) was covalently coupled to 500uL batch size Quanterix 750nM beads (catalog number 103529) using the Simoa Homebrew Assay Starter Kit (Catalog 101351). After antibody-bead conjugation was completed, the Simoa Quanterix HD-X analyzer was programmed to run a fully automated 3-step digital assay in the following manner:

First, antibody coated paramagnetic beads are incubated with calibrators of known concentration and specimens of unknown concentrations. The calibrator used for this assay is R&D Systems Recombinant Cathepsin L Protein, part number 842813 supplied as part of the DuoSet catalog DY952.

After undergoing a wash cycle, the beads and captured analyte are incubated with biotinylated detection antibody. The detection antibody used for this assay is R&D Systems Human Cathepsin L Biotinylated Antibody, (part 842812, catalog number DuoSet DY952).

After undergoing a second wash cycle, the beads/analyte/biotinylated antibody complex is incubated with the enzyme conjugate, streptavidin β-galactosidase (SBG) & substrate, β-D-galactopy-ranoside (RGP). The bead complex with captured analyte is introduced to a proprietary disc with micro-wells large enough for 1 bead complex, via a slight vacuum. Oil is delivered to the array via the same channel, removing excess beads from the surface of the array and sealing the loaded wells. The instrument camera images the sealed wells, capturing the fluorescence emitted by the enzymatic product of the labelled immunocomplexes. The instrument analyzes the image, determines the average enzymes/bead (AEB), and generates a calibration curve and sample concentration back calculated via 4-parameter logistic regression using the values from the calibration curve.

Human ICAM-1 was quantified using the MSD V-Plex Vascular Injury Panel 2 (Catalog K151SUD). The assay was performed in accordance with kit instructions. The Vascular Injury Panel 2 is supplied with anti-human ICAM-1 antibodies immobilized onto a carbon fiber spot in each well. Other analytes are available to run in multiplex format, however this assay was performed with only ICAM-1. The assay is performed by diluting samples 500x and adding them to appropriate wells. QC material and Calibrator are also added. The samples incubate at room temperature on a plate shaker for 2 hours. After the incubation, the plates are washed to remove any unbound substances. Detection Antibody Solution, which is labeled with MSD SULFO-Tag, is added, and incubated for 1 hour. After the incubation, the plates are then washed a final time and Read Buffer is added to each well. The plates are then read on the MSD Quick Plex 120 analyzer where the analyzer applies an electrical charge to each well. The electrical signal causes a chemical reaction where light is emitted from each spot and well. The amount of light is back calculated against known concentrations of ICAM-1 in the calibrator and quantified using 4 parameter logistic regression.

Human D-Dimer was quantified using the ProcartaPlex Multiplex Immunoassay Simplex kit, catalog number EPX01A-12149-901. The assay was run in accordance with the kit instructions. ProcartaPlex Immunoassays use microsphere (bead) technology developed by Luminex Corporation. Human serum or plasma, QC and calibrator are incubated with anti-human-D-Dimer coated paramagnetic beads, which capture any human D-Dimer present in the sample. After an incubation period, the beads are bound to the plate using a strong magnet and washed to remove any unbound substances. After the wash, the plate is removed from the magnet and biotinylated detection antibody is added to each well and incubated for 30 minutes. After the incubation the plate is again introduced to the magnet and washed again to remove any unbound substances. After the wash, the plate is removed from the magnet and Streptavidin-Phycoerythrin is added to each well. The plate is then incubated for 30 minutes. After the incubation, the plate is again introduced to the magnet and washed to remove unbound particles. After the wash, the plate is removed from the magnet and reading buffer is then added to each well. After a short incubation on a plate shaker to resuspend the magnetic beads, the plate is read on a Luminex 200 analyzer. Through hydrodynamic focusing, 2 lasers on the Luminex 200 assess the quantity of each dye-en-coded bead, and the quantity of fluorescence emitted by the Phycoerythrin. The concentration of each sample is back calculated to the known concentration of the calibrator using 5-parameter logistic regression.

### Multiplexed Autoantibody Profiling Using Bead-Based Protein Arrays

For a subset of samples, we analyzed autoantibody levels against a large panel of proteins, including type 1 interferons.

#### Bead-based antigen arrays

A custom bead-based antigen array was created, as previously described [14, 15]. A complete list of all antigens, vendors, and catalogue numbers can be found in Supplementary Table 1. The array included 58 commercial protein antigens, including cytokines, chemokines, growth factors, acute phase proteins, and cell surface proteins. Antigens were coupled to carboxylated magnetic beads (MagPlex-C, Luminex Corp.), each with a unique barcode [16, 17]. Commercially available mouse monoclonal antibodies or antibodies specific for engineered epitope tags were used to confirm the proper immobilization of some antigens to the beads. Prototype human plasma samples with known reactivity patterns were used to validate the conjugated beads. Validated beads were combined to form the final antigen array.

#### Array probing

Serum or plasma samples were diluted at 1:100 in 0.05% PBS-Tween supplemented with 1% (w/v) bovine serum albumin and transferred into 96-well plates. The bead array was distributed into a 384-well plate (Greiner BioOne) by transfer of 5 µL of bead array per well. Then 45 µL of the 1:100 diluted sera were transferred into the 384-well plate containing the bead array. Samples were incubated for 60 minutes on a shaker at room temperature. Beads were washed 3 times with 60 µL PBS-Tween on a plate washer (EL406, Biotek), and 50 µL of 1:1000 diluted R-phycoerythrin (R-PE) conjugated Fc-γ-specific goat anti-human IgG F(ab’)2 fragment (Jackson ImmunoResearch) was added to the 384-well plate for detection of bound human IgG. After a 30-minute incubation, the plate was washed 3 times with 60 µL PBS-Tween and re-suspended in 50 µL PBS-Tween prior to analysis using a FlexMap3D™ instrument (Luminex Corp.). Binding events were displayed as Mean Fluorescence Intensity (MFI). All samples were run in duplicate in each experiment. A static cutoff of 3000 MFI was used to determine positive autoantibody reactivities.

### Statistical Analysis

Continuous variables are presented with mean and range. Group differences in demographics were evaluated using chi-squared tests for categorical variables and 2-way analysis of variance (ANOVA) for continuous variables. Correlation analysis between antibody response and cytokine level was performed using Spearman’s correlation. All statistical analyses and scientific graphics were made by using GraphPad Prism 8.0 (GraphPad Software, Inc, CA).

## RESULTS

### Study Cohort

A total of 230 (181 unique patients) samples were analyzed across the 5 COVID-19 disease severities: PCR-negative mild, PCR-positive mild, PCR-positive moderate, PCR-positive severe, and pre-pandemic samples. Amongst the PCR-positive disease groups, samples were obtained from patients ranging in age from 19-89 years, with a mean age in each group of 41.17 (range, 19-67), 56.63 (range, 22-80), and 56.43 (range, 22-89) years respectively. A total of 49 samples from female participants and 42 from male participants were analyzed within the PCR-positive groups, with 22 (44.89%) females and only 9 (21.3%) males in the mild disease group compared to 15 (30.6%) females and 15 (35.7%) males in the moderate disease group, and 12 (24.5%) females and 18 (42.8%) males in the severe disease group. A comprehensive break down of available demographic details is shown in Table 2.

**Table 2.** Participant Demographics

	Pre-Pandemic Negative Controls	PCR-Negative Symptomatic Mild	PCR-Positive Symptomatic Mild	PCR-Positive Symptomatic Moderate	PCR-Positive Symptomatic Severe
N	60	30	31	59	50
N (independent)	60	30	31	30	30
Average Age (yrs)			41.17	56.63	56.43
Age Range (yrs)			19–67	22–80	22–89
Females			22	15	12
Males			9	15	18
Race: White			16	4	6
Race: Black or African American			4	19	13
Race: Hispanic or Latino			3	4	8
Race: Asian			2	2	2
Race: Other/Not Documented			4	1	1
Death			0	2	9

Available demographic information across disease severities, including age, sex, race/ethnicity, and mortality outcomes.

** No demographic information, including age, sex, or race/ethnicity were available for the pre-pandemic negative control group or PCR-negative mild group due to the retrospective nature of this study and participant sample records.

### Antibody Response Characteristics Change Across Disease Severity

**Figure 1.**
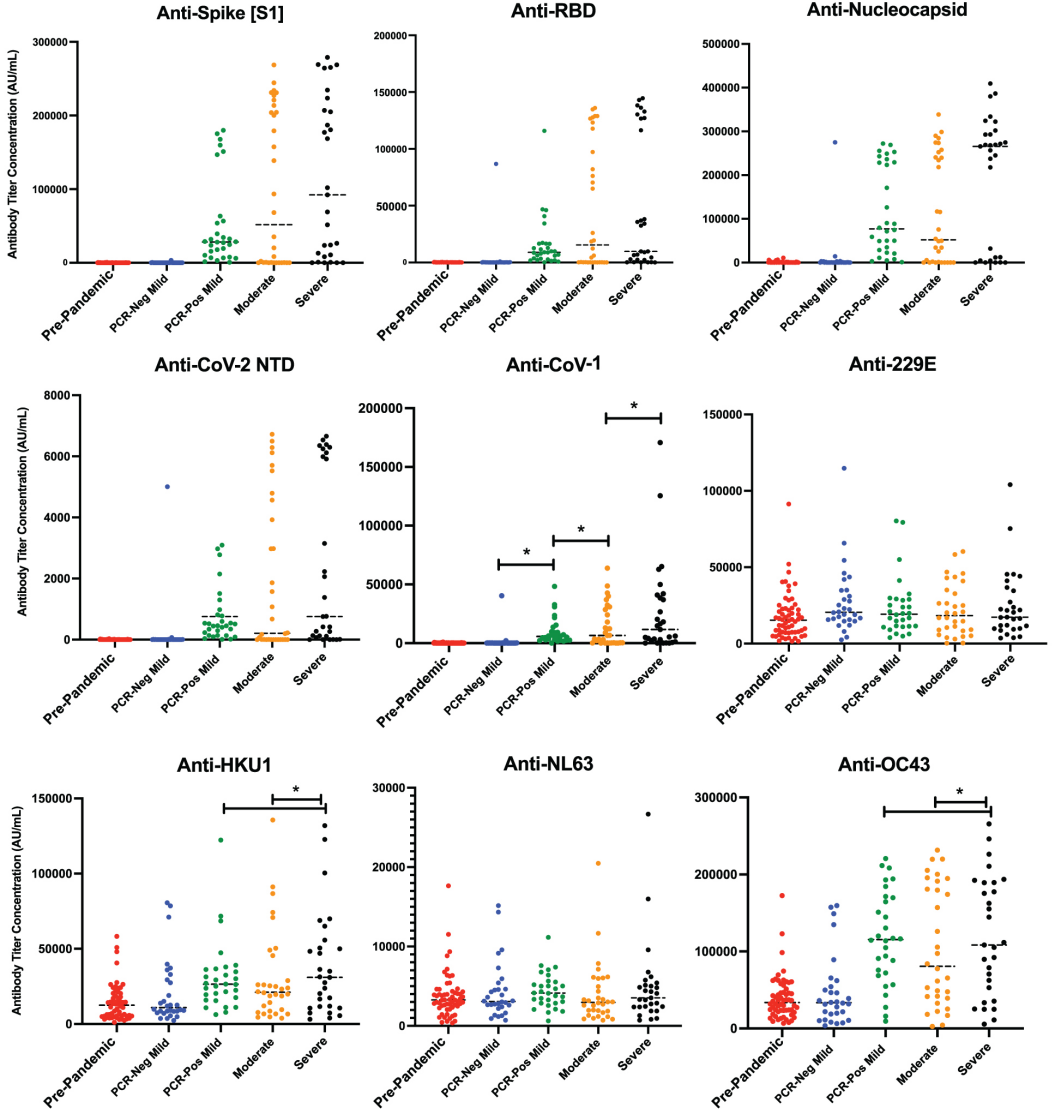
**Quantitative antibody response by disease severity**. Dot plot representation of SARS-CoV-2 antibody concentration against spike (A), RBD (B), nucleocapsid (C), and N-terminal Domain of the nucleoprotein (NTD, D) across disease cohorts. Dot plot representation of samples for antibody cross-reactivity against the spike protein of SARS-CoV-1 (E), along with other alpha and beta coronaviruses (F-I) across disease severity. A static cutoff of 1000 AU/mL (anti-spike), 800 AU/mL (anti-RBD), and 5000 AU/mL (anti-N), depicted by the gray dashed line, was used to determine positive antibody response. SARS-CoV-2 PCR-positive mild, moderate, and severe disease cohorts had increased antibody titers against CoV-1, with increasing antibody concentration by increased disease severity (*P* < 0.001). HKU1 and OC43 anti-S concentrations were statistically significantly increased in severe disease groups, compared to mild and moderate disease groups (*P* < 0.001). *: *P* < 0.001

Using the MSD COVID-19 Serology kit, which included epitopes from SARS-CoV-2 and alpha and beta coronavirus species, we screened samples for their relative concentrations of IgG antibodies against target viral antigens. As can be seen in Figure 1, antibody response varied by both disease severity, and within each severity group there was heterogeneity in serologic response.

Positive and negative antibody response cutoff values were estimated based on pre-pandemic, SARS-CoV-2 naive, samples. A positive antibody response for all SARS-CoV-2 antigens was defined as AU/mL values above 1,000 AU/mL for the spike (S1) epitope, 800 AU/mL for RBD, and 5,000 AU/mL for nucleocapsid. Among the PCR-positive mild samples, 30/31 (96.7%) showed a positive anti-spike antibody response (median AU/mL: 28,544.24 [CI: 17,712.23 – 53,830.29]).

However, only 18/30 (60%; median AU/mL: 202,028.9 [CI: 93,406.05 – 227,184.6]) individuals in the moderate disease group and 24/30 (80%; median AU/mL: 177,045.0 [CI: 51,445.99 – 223,582.3]) in the severe disease group demonstrated a positive anti-spike antibody response at hospital admission. Four (3 moderate, 1 severe) patients never developed a positive anti-spike response upon subsequent sampling between 8 to 34 days after initial sampling. The rest of the samples, including the remaining 9/30 moderate and 5/30 severe samples later developed a positive antibody response on subsequent sampling 7 to 10 days later (Table 3). Similar antibody responses were seen for anti-RBD and anti-nucleocapsid, with the mild disease group showing a positive antibody response of 29/31 (93.5%) and 28/31 (90.3%), respectively. The same patient samples that demonstrated an initial lack of antibody response to anti-spike, also showed a delayed or lack of response for anti-RBD and anti-nucleocapsid antibodies. Similarly, the same 4 patients who never developed an anti-spike response, also did not develop an anti-RBD or anti-nucleocapsid response.

**Table 3.** Longitudinal Quantitative Antibody Data on a Subset of PCR-positive Moderate and Severe Disease Cohort Samples

	Initial Sample N(%) Seropositivity	Sample 2 N(%) Seropositivity	Sample 3 N(%) Seropositivity
**Anti-Spike**			
Total # of samples	34	34	8
Moderate	7 (38%)	15 (83%)	3 (75%)
Severe	10 (62.5%)	15 (93.75%)	3 (75%)
**Anti-RBD**			
Total # of samples	34	34	8
Moderate	6 (33.3%)	14 (77.78%)	3 (75%)
Severe	10 (62.5%)	15 (93.75%)	3 (75%)
**Anti-Nucleocapsid**			
Total # of samples	34	34	8
Moderate	9 (50%)	14 (77.78%)	3 (75%)
Severe	9 (56.25%)	14 (87.5%)	2 (50%)

Subsequent sampling was performed 7 to 10 days following initial sample collection to assess for antibody seropositivity against spike, RBD, and nucleocapsid. Seropositivity was defined based on validation data described in Table 1, with anti-spike >1000 AU/mL, anti-RBD >800 AU/mL, and anti-nucleocapsid >5000 AU/mL).

In assessing cross-reactive antibody response to spike protein amongst other alpha and beta coronaviruses during acute SARS-CoV-2 infection, increased antibody titers were seen against CoV-1 amongst all 3 PCR-positive groups compared to pre-pandemic samples, where there was increasing antibody concentration with increased disease severity (*P* < 0.001) (Figure 1).

In comparing disease severity groups, the mean antibody titer to CoV-1 during acute SARS-CoV-2 infection was 9775.385 AU/mL in the mild group, 14678.00 AU/mL in the moderate group, and 26628.01 in the severe group. A statistically significant increase in HKU1 and OC43 anti-S concentration was only observed amongst the severe disease groups, with mean titers of 38678.42 AU/mL and 119344.0 AU/mL, respectively, compared to 30867.16 and 38678.42 in the HKU1 mild and moderate disease groups (*P* < 0.001), and 117573.1 AU/mL and 105053.4 AU/mL in the OC43 mild and moderate disease groups (*P* < 0.001).

No statistically significant change was seen in anti-S titer across disease groups in 229E and NL63 (*P* > 0.05) [13]. Given the variability in response or lack thereof between anti-S titer concentrations between alpha and beta coronaviruses, the presence or production of cross-reactive antibodies could not be adequately concluded to play any significant role in disease severity.

Based on these results, we wanted to better understand whether the high responders seen in anti-S1 serology analysis, or those whose antibody titers were above the mean titer at baseline, correlated with the high responders seen in serologic assays for various alpha and beta coronavirus species as a marker of disease severity. For individual patient samples, we thus analyzed the anti-spike IgG response at patients’ initial visits for various alpha and beta coronavirus species.

A total of 49 longitudinal samples amongst the PCR-positive moderate (n=29) and severe (n=20) disease groups were available, in which analysis of patients’ humoral responses was evaluated. No longitudinal data was obtained among the PCR-negative mild or PCR-positive mild disease groups. Between the PCR-moderate and severe groups, a total of 18 and 16 patients, respectively, had at least 1 follow-up serologic sample taken at least 7 days after the baseline sample. In most patients, an increase in antibody concentration between the initial sample and any subsequent samples across both moderate and severe disease and against each coronavirus epitope (S, RBD, N) was observed, with the mean number of days between the first and last samples being 8.1 days. Only 2 patients in the moderate disease group and 2 patients in the severe disease group saw a decrease in antibody titer concentration, with the average number of days between the first and last samples being 57.25 days.

**Figure 2.**
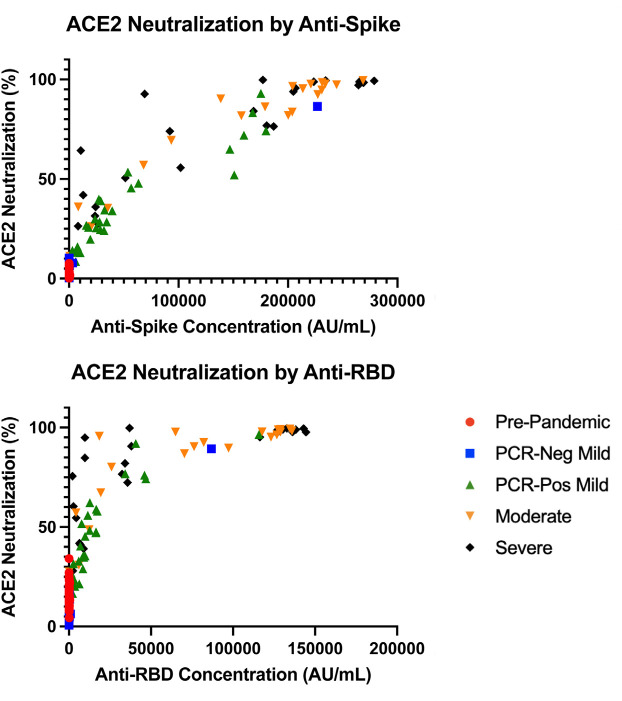
**Analysis of ACE2 inhibition by anti-S1(A) and anti-RBD (B) IgG antibodies by disease severity.** Quantitative plasma anti-S had a correlation of r = 0.885 (*P* < 0.001) and anti-RBD had a correlation of r = 0.75 (*P* < 0.001) to ACE2 neutralization.

To assess whether the difference in antibody quantity seen across disease severity and within disease groups correlated with antibody function, we assessed the antibody blocking ability by disease severity and antibody response (Figure 2). Interestingly, antibody quantity directly correlated with functional ability to block virus binding to membrane bound ACE2, where a lower SARS-CoV-2 anti-spike/anti-RBD response corresponded with a lower antibody blocking potential compared to higher antibody response (anti-S1 r = 0.884, *P* < 0.001; anti-RBD r = 0.75, *P* < 0.001). However, the degree of correlation between antibody quantity and antibody function showed a statistically significant decrease amongst the severe disease group compared to mild and moderate disease groups, where the mild and moderate disease groups had an r = 0.82 and 0.86, respectively, compared to the severe disease group with an r = 0.74 (*P*<0.05), though all PCR-positive groups still had statistically significant correlations (*P* < 0.0001).

### Plasma Cytokine Levels Indicate Disease Severity, But Not Antibody Response

**Figure 3.**
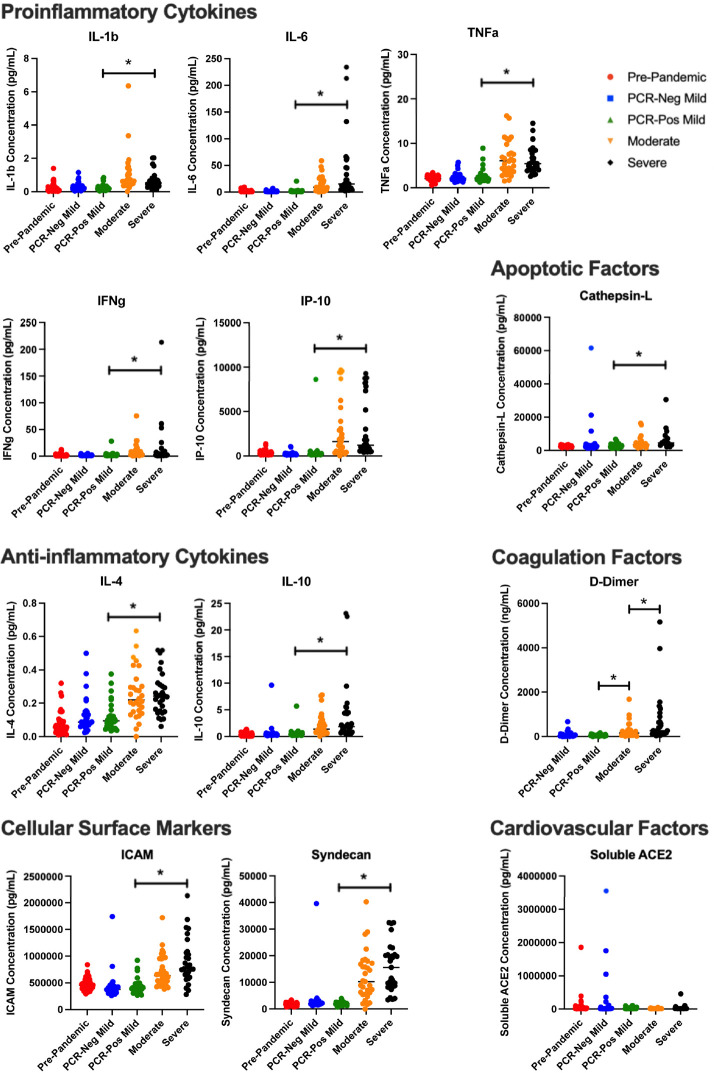
**Inflammatory markers across disease severity.** Dot plot representation of cytokine, coagulation factors, cell surface markers, cardiovascular and apoptotic factors, across disease severity cohorts. IFNγ, TNFα, IL-1β, IL-4, IL-6, IL-10, IP-10, ICAM, Syndecan, Cathepsin-L, and D-Dimer statistically significantly differed between PCR-positive non-hospitalized (mild disease) and PCR-positive hospitalized (moderate and severe disease) cohorts (*:*P* < 0.05). D-Dimer also significantly differed among hospitalized moderate and severe COVID-19 disease groups (*P* < 0.05).

Plasma samples were evaluated to determine the relative quantity of proinflammatory cytokines and immune mediators present at the time of symptomatic infection to determine whether cytokine level correlated with antibody response. As was expected based on progressive symptomatic disease presentation, proinflammatory cytokine microenvironment and endothelial marker response exhibited a positive correlation with increasing disease severity. Of the 12 soluble plasma markers analyzed, 11 were statistically significantly different between PCR-positive non-hospitalized (mild disease) and PCR-positive hospitalized (moderate and severe disease), with all showing an increasing quantity by disease severity, including IFNγ, TNFα, IL-1β, IL-4, IL-6, IL-10, IP-10, ICAM, Syndecan, Cathepsin-L, and D-Dimer (*P* < 0.05; Figure 3). Of those 11 cytokines, only D-Dimer significantly differed among hospitalized moderate and severe COVID-19 disease groups (*P* = 0.028).

Of the plasma markers measured, soluble ACE2 (sACE2) was the only one that did not show a statistically significant correlation between PCR-positive disease groups (*P* > 0.1). However, there was a statistically significant decrease in sACE2 between the PCR-negative mild group and the 3 PCR-positive groups (*P* < 0.001). While there was no statistical significance, there was a quantitative difference between the PCR-positive mild, moderate, and severe disease groups, where the mean concentration of sACE2 was 26,631 pg/mL, 17,469 pg/mL, and 41,904 pg/mL, respectively.

**Figure 4.**
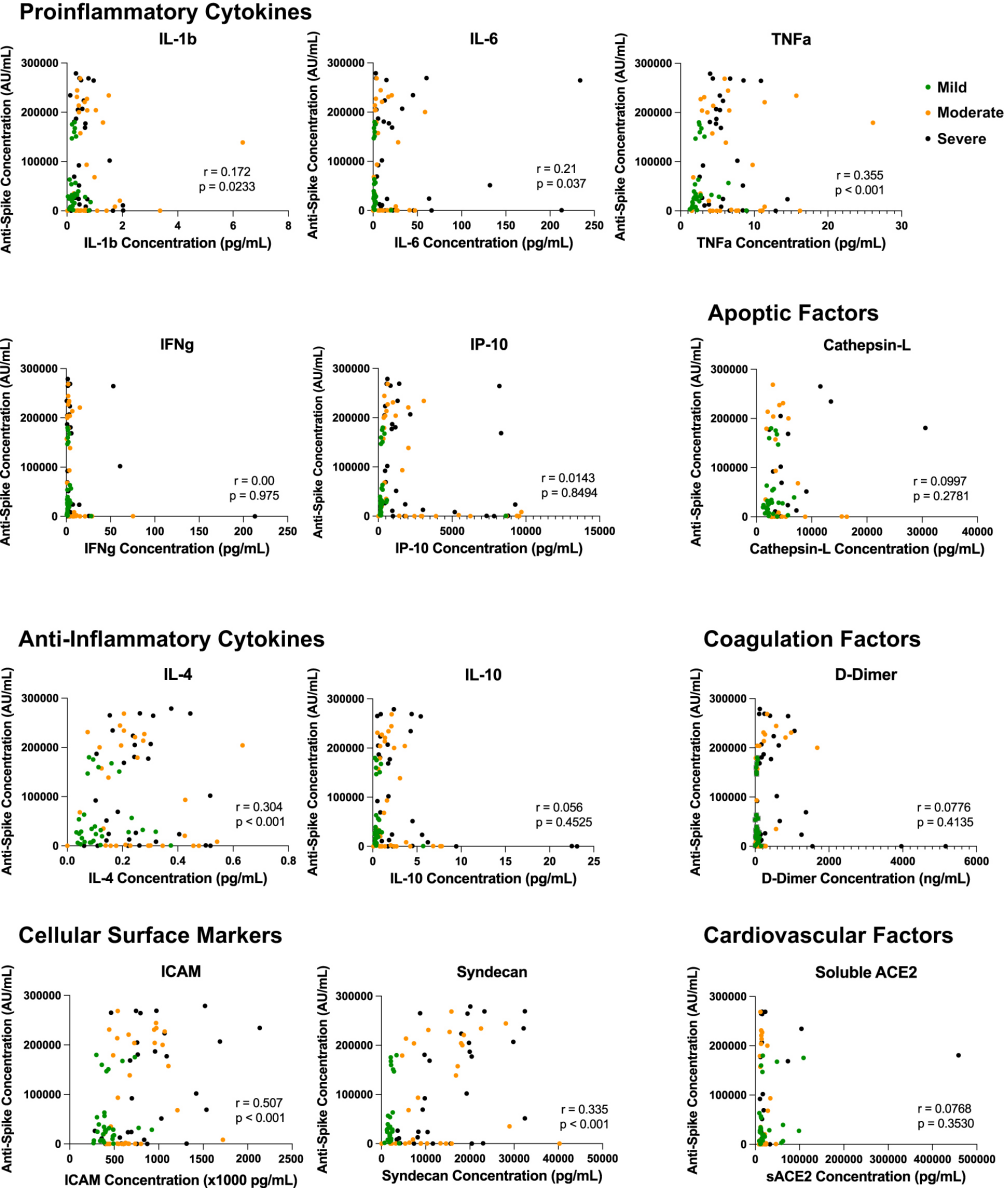
**Inflammatory dysregulation between cytokine microenvironment and antibody production.** Correlation analysis of inflammatory marker plasma concentration and anti-spike antibody production across disease severity. Across all soluble proinflammatory markers, 6 showed a statistically significant positive correlation between cytokine or epithelial marker and antibody quantity regardless of COVID-19 disease severity, including ICAM (*P* < 0.001), IL-1β (*P* = 0.0233), IL-4 (*P* < 0.001), IL-6 (*P* = 0.037), TNFα (*P* < 0.001), and Syndecan (*P* < 0.001).

**Figure 5.**
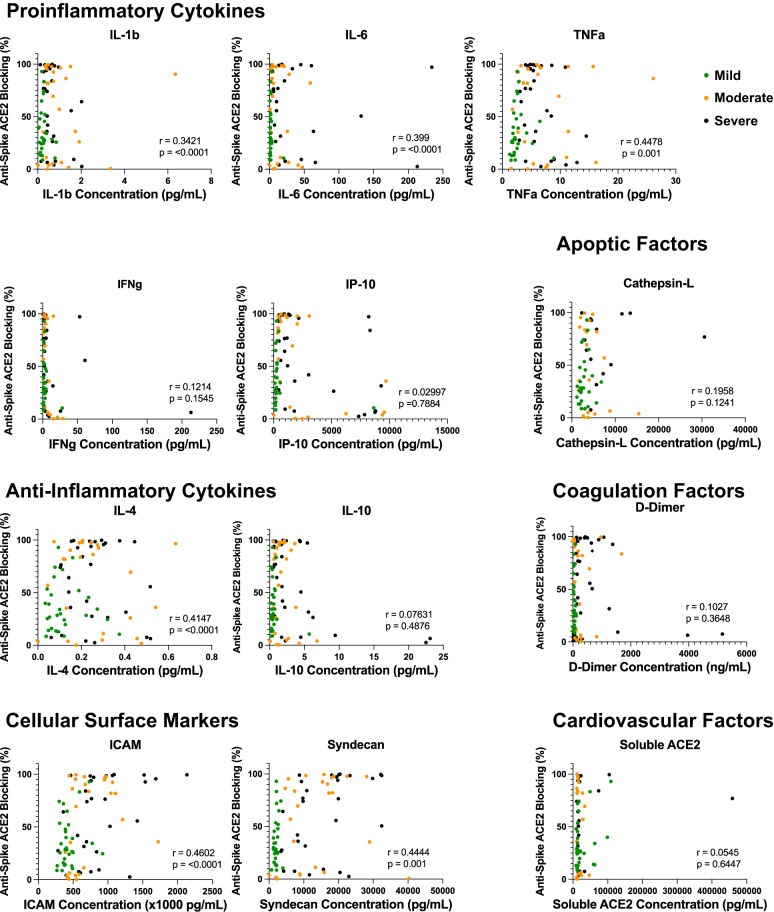
**Proinflammatory dysregulation between cytokine microenvironment and antibody neutralization capacity.** Correlation analysis of inflammatory marker plasma concentration and anti-spike antibody ACE2 neutralization disease severity. Elevations in ICAM (*P* < 0.0001), IL-1β (*P* = 0.0233), IL-4 (*P* < 0.0001), IL-6 (*P* = 0.0001), TNFα (*P* = 0.001), and Syndecan (*P* = 0.00) all showed statistically significant correlations to antibody neutralization capacity amongst PCR-positive COVID-19 disease cohorts.

To assess whether these increased soluble cytokine concentrations correlated with the increase in antibody quantity and/or blocking capacity, we performed a Spearman’s correlation to better understand any potential correlative relationship (Figures 4 and 5). Across all soluble proinflammatory markers we examined, 6 showed a statistically significant positive correlation between cytokine or epithelial marker and antibody quantity regardless of COVID-19 disease severity (Figure 4), including ICAM (r = 0.507, *P* < 0.001), IL-1β (r = 0.172, *P* = 0.0233), IL-4 (r = 0.304, *P* < 0.001), IL-6 (r = 0.21, *P*= 0.037), TNFα (r = 0.335, *P*< 0.001), and Syndecan (r = 0.613, *P* < 0.001). Similar results were seen between cytokine or epithelial marker and antibody blocking capacity regardless of COVID-19 disease severity (Figure 5). This same correlation did not exist within disease severity groups for these 6 cytokines, with all plasma markers showing no statistically significant correlation (*P* > 0.05). All other serologic markers showed no correlation between antibody quantity or blocking ability across disease severity (*P* > 0.05).

### Autoantibody Findings in a Subset of Samples

Autoantibodies are commonly observed in patients with SARS-CoV-2 infection, particularly patients who are severely ill. Moreover, approximately 25% of hospitalized COVID-19 patients develop at least 1 new-onset autoantibody, suggesting SARS-CoV-2 has the capacity to trigger the development of autoantibodies and potentially autoimmune manifestations. We used the same protein microarray platform used recently to study SARS-CoV-2 [18] and more recently in patients with non-SARS-CoV-2 pulmonary infections [19] to profile autoantibodies against native protein antigens. Array data will be uploaded to the GEO database upon publication of this manuscript.

**Figure 6.**
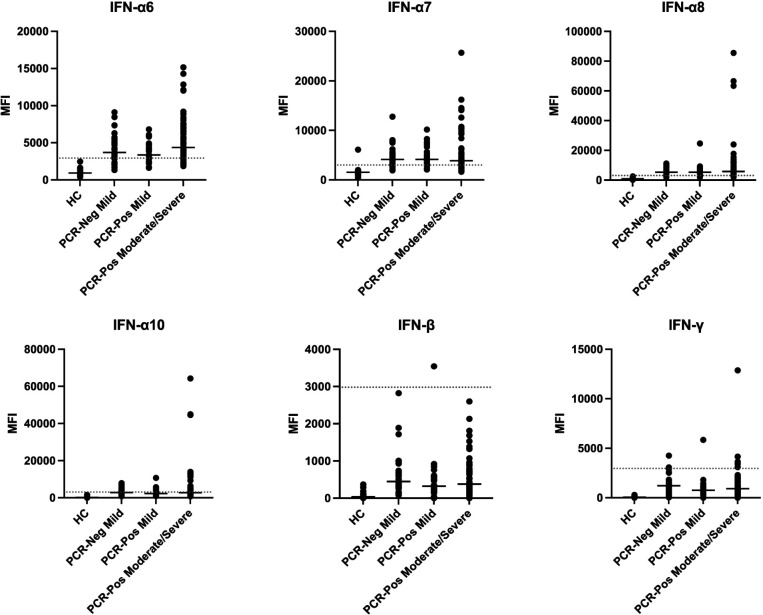
**Prevalence of anti-interferon antibodies in a subset of patients.** Plasma IgG antibodies were profiled with a 58-plex bead-based protein array containing cytokines and chemokines. Example distributions of autoantibodies against interferons in healthy controls (HC, n = 17), PCR-negative mild patients (n = 31), PCR-positive mild patients (n = 27), and PCR-positive moderate/severe patients (n = 60) are shown. MFI is shown on the y-axis. A static cutoff of 3000 MFI, depicted by the gray dashed line, was used to determine positive anti-interferon response.

We focused our analysis on anti-interferon autoantibodies since these have been shown to be pathogenic, strongly correlating with worse clinical outcomes in COVID-19 [20]. Although anti-interferon autoantibodies were not statistically significant between groups possibly due to small sample sizes, we identified multiple SARS-CoV-2 infected patients with very high levels of anti-interferon autoantibodies, particularly Type I interferons 6, 7, 8, and 10 (Figure 6). Based on our static cutoff of 3000 MFI to determine positive autoantibody reactivities, clear, positive antibody responses against Type I IFN-β were not detected (Figure 6). One patient was identified with anti-IFN-γ autoantibodies, which have been observed in SARS-CoV-2 infection but lack receptor blocking activity [18, 20].

## DISCUSSION

This study presents a comprehensive analysis of antibody response associated with COVID-19 disease severity amongst a cohort of 181 unique patient samples taken at the start of the COVID-19 global pandemic, including 60 pre-pandemic samples obtained between 2004 and 2014. Using the Mesoscale multiplex assay system, we were able to analyze both the quantity and functional capacity of the antibody response against SARS-CoV-2 infection across all patient samples, correlating response rates and relative concentrations of circulating inflammatory markers to better understand the differences seen in the humoral response at the time of active acute infection across disease severities.

Our study demonstrated that both antibody production and function increased with more severe COVID-19 disease, where increased antibody concentration was positively correlated with increased antibody blocking function across disease states. We also found that proinflammatory cytokines IL-1β, IL-4, IL-6, and TNFα, along with endothelial markers ICAM and Syndecan, were positively correlated with both quantitative and qualitative antibody production, indicating a possible role for these cytokines in predicting humoral immune response.

Similar to SARS-CoV-1 infection, SARS-CoV-2 is known to produce a dysregulated and hyperinflammatory immune response, resulting in severe morbidity and mortality [21]. Previous studies have shown that increased viral load is characteristic of disease progression in COVID-19, following initial infection [5]. As is true of other infectious agents, as SARS-CoV-2 viral load increases, so does the immune response against the virus. This immunologic picture is best thought to represent the mechanism of increasing antibody response seen in more severe COVID-19 disease, as numerous other studies have reported increasing quantitative humoral immune response with disease progression [6–8, 22]. Our data demonstrates these same results, where increased disease severity correlated with an enhanced quantitative antibody response and blocking function, such that antibody concentration is highly correlated with antibody function.

To better understand this correlative relationship between disease severity and humoral immune response, many have proposed the role of cross-reactive anti-spike antibodies from other alpha and beta coronaviruses as a potential mediator in early SARS-COV-2 viral recognition and clearance, and a mechanism for prevention of severe COVID-19 disease [23]. It was thought that antibodies against homologous epitope regions amongst the seasonal coronaviruses and SARS-CoV-2 may aid in faster viral clearance, rapidly decreasing viral load and preventing disease progression, while also resulting in decreased SARS-CoV-2 specific humoral immunity. While early studies found elevated antibody titer levels to seasonal coronavirus post SARS-CoV-2 infection, more recent literature remains inconclusive regarding the role in cross-reactive antibodies [24–26]. Likewise, our results demonstrated an overall increase in antibody production across coronavirus species. However, with no clear pattern or correlation of anti-spike antibody response between individual coronaviruses and disease severity, it is diﬃcult to determine whether or not recent seasonal coronavirus infection might aid in or mitigate the inflammatory response and/or clinical course of SARS-CoV-2 infection.

We characterized mediators of the immune response and markers of inflammation to help delineate the humoral immune response seen across disease severity. IL-6 is an important cytokine released by macrophages in response to antigen recognition leading to a proinflammatory acute phase immune response thought to play an important role in cytokine storm and severe SARS-CoV-2 infection [10, 12]. High levels of IL-1β, IL-6, IP-10, and TNFα have been associated with acute respiratory distress syndrome (ARDS), severe disease, and mortality following SARS-CoV-2 infection [10–13, 27]. Recent studies have also shown that proinflammatory cytokines, such as IL-6, IL-8, and TNFα show a positive correlation with SARS-CoV-2 IgG antibody production [28]. Similarly, we found that levels of IL-1β, IL-4, IL-6, and TNFα not only increased with severe disease, but also correlated with the adaptive immune response both quantitatively and qualitatively, leading to increased blocking of the virus to bind the ACE2 receptor for cell entry. While IFNγ, IL-4, IL-10, and IP-10 did not seem to directly correlate with antibody response amongst our cohorts, they were elevated in moderate and severe COVID-19 disease, compared to mild infection.

Increased circulating levels of ICAM and Syndecan are highly associated with endothelial damage and inflammation. Previous studies have found that Syndecan is a useful biomarker for monitoring disease progression and organ damage in critically ill patients under various settings [29, 30]. Changes in plasma Syndecan levels significantly correlated with severe disease progression and mortality among patients infected with SARS-CoV-2 [30]. Similarly, ICAM has been found to be significantly elevated in early phases of COVID-19-related ARDS, which often leads to subsequent severe disease [31]. Like the cytokines mentioned previously, both Syndecan and ICAM were found to correlate with increased antibody production and function following SARS-CoV-2 infection. This is mostly likely explained by the hyperinflammatory state of progressive severe disease. While other markers of endothelial damage and coagulopathy, such as D-Dimer, were not found to be significantly correlative of antibody response, they were still elevated in patients with severe disease compared to uninfected and patients with mild COVID-19 disease, indicating a larger immunologic and pathophysiologic response that is still yet to be defined.

Interestingly, sACE2 has become a marker of interest in the pathogenesis of the SARS-CoV-2 virus. As is well documented, the SARS-CoV-2 virus binds to the ACE2 receptor on the membrane of epithelial cells (mACE2) found in the lungs, gastrointestinal tract, heart, and testis. Specifically, it has been shown that once the receptor binding domain of the spike protein found on the surface of the SARS-CoV-2 virus is bound to the enzymatic portion of the mACE2, it results in endocytosis and translocation of both the virus and enzymes of the endosome into the host cell, resulting in infection [32]. Soluble ACE2 (sACE2), acts similarly in the renin-angiotensin-aldosterone signaling (RAAS) pathway as mACE2, and has been hypothesized to have a protective effect against SARS-CoV-2 virus induced lung injury, as well as viral host cell entry and replication. It is thought that increasing levels of sACE2 in the blood stream leads to increased levels of vasodilator angiotensin-(1-7), resulting in decreased injury to the lung parenchyma, decreased COVID-19 disease severity, and better outcomes [33]. Similarly, it is also hypothesized that free and soluble ACE2 may also bind to the spike protein of SARS-CoV-2, rendering those coronavirus spikes unavailable for binding to mACE-2 sites, effectively neutralizing the virus [34]. Our results show that while there was not a statistical difference between the decrease in sACE2 between PCR-positive mild and moderate groups, the decline in circulating levels may have led to increased clinical severity. Interestingly however, the severe cohort saw an almost 2-fold increase in circulating sACE2 levels, making this same inference unlikely. It can be inferred that the steep rise in sACE2 levels among the severe disease cohort is most likely due to immune dysregulation and possible comorbid factors, including preexisting cardiovascular disease and diabetes, both of which are known to increase circulating sACE2 baseline levels, as well as lead to more severe COVID-19 disease.

Interestingly, a subset of patients who required inpatient hospitalization for moderate or severe disease and who mounted a significant antibody response to the SARS-CoV-2 virus exhibited high levels of titers [23] to circulating type 1 interferons, compared to other participants. Other studies have shown similar results, where high levels of type 1 interferon autoantibodies were reported in 10% to 20% of COVID-19 patients with severe disease [35–38]. In this study, the authors suggested that pre-existing anti-type I interferon IgG with receptor blocking activity were pathogenic and indicated patients who were more likely to go on to develop severe disease. High levels of anti-cytokine autoantibodies including anti-type I IFN have recently been shown to characterize ICU patients infected with other pathogens, including influenza [22]. Taken together, these studies suggest that not only does this immune-mediated response indicate a hyper-reactivity to the virus, but these laboratory findings may be a good predictor of severe disease in those presenting to the hospital.

Our study has several limitations. First, this was a retrospective study based on random sampling and availability of plasma samples from the biorepository at Rush University Medical Center. Second, comprehensive clinical data and longitudinal sampling was not available, including comorbid factors, that might have contributed to disease severity or humoral immune responses.

Previous studies have shown that proinflammatory markers, including IL-6, IL-8, IL-1β, and TNFα, are significant predictors of COVID-19 disease severity, regardless of demographics or comorbidities. Our study demonstrated that not only are these proinflammatory markers, as well as IL-4, ICAM, and Syndecan, correlative of disease severity, they are also correlative of antibody quantity and quality following SARS-CoV-2 infection. These data have important implications, not only in the correlation of disease state, but also to the SARS-CoV-2 immunologic response and subsequent long-term humoral immunity. Further studies are needed to assess how these data may relate to vaccine response, repeat SARS-CoV-2 infection, and long-COVID.

## SUPPLEMENTARY DATA

Supplementary materials are available at the *Pathogens and Immunity* website. Supplementary data may be provided by the authors to benefit the reader. Supplementary data are not copyedited and are the sole responsibility of the authors. Questions or comments related to supplementary materials should be addressed to the corresponding author.
