# Supplementary material for: Immune Dysregulation in Acute SARS-CoV-2 Infection
Source: Pathog Immun. 2023 Feb 20;7(2):143–70. doi: 10.20411/pai.v7i2.537 (PMC9973727; doi:10.20411/pai.v7i2.537)
Supplement: Supplementary Table 1 [file pai-7-143-s01.pdf]

PATHOGENS AND IMMUNITY
IN ASSOCIATION WITH CASE WESTERN RESERVE UNIVERSITY AND THE RICHARD J. FASENMYER FOUNDATION

SUPPLEMENTAL TABLE 1 - CYTOKINE ARRAY CONTENT

Bead ID	Antigen	Vendor	Catalog #
1	Bare Bead		
2	Human IgG from serum	Sigma	I4506
3	Anti-Human IgG Fc fragment specific	Jackson	109-005-008
4	Anti-Human IgG (H+L)	Jackson	109-005-003
5	Anti-Human IgG F(ab') fragment specific	Jackson	109-005-006
6	CD74	Prospec	PRO-1467
7	IFNλ2	Peprotech	300-02K
10	IL-1α	Prospec	CYT-253
13	IFNα1	Prospec	CYT-291
14	IFNα7	Prospec	CYT-196
16	IFNα10	Sino Biological	10349-H08H
18	IFNα8	Sino Biological	10347-H08H
19	IFNα6	Origene	TP760329
20	IL-2	Peprotech	200-02
21	IL-4	Peprotech	200-04
22	IL-13	Peprotech	200-13
23	IL-21	Peprotech	200-21
24	Fractalkine/CX3CL1	Peprotech	300-31
25	IP-10/CXCL10	Peprotech	300-12
26	IL-31	Prospec	CYT-625
27	IL-6	Prospec	CYT-098
28	MCP-2/CCL8	Peprotech	300-15
29	OSM	Peprotech	300-10
30	IL-11	Prospec	CYT-214
31	SDF-1a/CXCL12	Peprotech	300-28A
32	IL-27	Prospec	CYT-048
33	CNTF	Prospec	CYT-272
34	CT-2	Prospec	PRO-1578
38	GM-CSF	Peprotech	300-03
39	IFNα2	R&D	11101-2
40	IFNβ	Peprotech	300-02BC

Grimm L, Onyeukwu C, Kenny G, Parent DM, Fu J, Dhingra S, Yang E, Moy J, Utz PJ, Tracy R, Landay A. Immune Dysregulation in Acute SARS-CoV-2 Infection. *Pathogens and Immunity*. 2023;7(2):143–170. doi: 10.20411/pai.v7i2.537.

41	IFNγ	Peprotech	300-02
42	IFNε	R&D	9667-ME-025/CF
43	IFNλ1	Peprotech	300-02L
44	IFNλ3	R&D	5259-IL-025/CF
45	IFNω	R&D	11395-1
46	IL-10	Peprotech	200-10
47	IL-12p40	Peprotech	200-12P40
48	IL-12p70	Peprotech	200-12
49	IL-15	Peprotech	200-15
50	IL-17F	Peprotech	200-25
51	IL-1β	Peprotech	200-01B
52	IL-22	Peprotech	200-22
55	TNFα	Peprotech	300-01A
56	TNFβ	Peprotech	300-01B
58	ACE2	Sino Biological	10108-H05H
59	Eotaxin	Peprotech	300-21
60	Eotaxin 2	Peprotech	300-33
62	IL-17A	Peprotech	200-17
63	IL-33	Peprotech	200-33
64	IL-7	Peprotech	200-07
65	MIP-1α	Peprotech	300-08
67	PDGFBB	Peprotech	100-14B
68	sRANK-ligand	Peprotech	310-01C
69	TIFIγ	Surmodics	A11001
70	CRP	Prospec	PRO-335
71	MPO	Prospec	ENZ-074
73	C3a	R&D	3677-C3-025
76	Gal-9	R&D	9064-GA-050
77	LIF	Peprotech	300-05
78	VEGFB	Peprotech	100-20B
79	HTRA1	R&D	2916-SE-020
80	d-dimer	LeeBio	200-13-0.1

Grimm L, Onyeukwu C, Kenny G, Parent DM, Fu J, Dhingra S, Yang E, Moy J, Utz PJ, Tracy R, Landay A. Immune Dysregulation in Acute SARS-CoV-2 Infection. *Pathogens and Immunity*. 2023;7(2):143–170. doi: 10.20411/pai.v7i2.537.
